# Errors linked to medication management in nursing homes: an interview study

**DOI:** 10.1186/s12912-021-00587-2

**Published:** 2021-04-29

**Authors:** Mariette Bengtsson, Ann-Britt Ivarsson Ekedahl, Karin Sjöström

**Affiliations:** Department of Care Science, Faculty of Health and Society, Malmö University, SE 205 06 Malmö, Sweden

**Keywords:** Delegation, Medication management, MTO concept, Nursing home, Safety

## Abstract

**Background:**

The number of errors in medication management in nursing homes is increasing, which may lead to potentially life-threatening harm. Few studies on this subject are found in the municipal nursing home setting, and causes need to be identified. The aim of this study was to explore perceptions of errors connected to medication management in nursing homes by exploring the perspective of first-line registered nurses, registered nurses, and non-licensed staff involved in the care of older persons.

**Methods:**

A qualitative research approach was applied based on semi-structured interviews with 21 participants at their workplaces: Seven in each of the occupational categories of first-line registered nurses, registered nurses, and non-licensed staff. Subcategories were derived from transcribed interviews by content analysis and categorized according to the Man, Technology, and Organization concept of error causation, which is as a framework to identify errors.

**Results:**

Mistakes in medication management were commonly perceived as a result of human shortcomings and deficiencies in working conditions such as the lack of safe tools to facilitate and secure medication management. The delegation of drug administration to non-licensed staff, the abandonment of routines, carelessness, a lack of knowledge, inadequate verbal communication between colleagues, and a lack of understanding of the difficulties involved in handling the drugs were all considered as risk areas for errors. Organizational hazards were related to the ability to control the delegation, the standard of education, and safety awareness among staff members. Safety issues relating to technology involved devices for handling prescription cards and when staff were not included in the development process of new technological aids. A lack of staff and the lack of time to act safely in the care of the elderly were also perceived as safety hazards, particularly with the non-licensed staff working in nursing homes.

**Conclusions:**

The staff working in nursing homes perceive that the risks due to medication management are mainly caused by human limitations or technical deficiencies. Organizational factors, such as working conditions, can often facilitate the occurrence of malpractice. To minimize mistakes, care managers need to have a systemwide perspective on safety issues, where organizational issues are essential.

## Background

Older people are often diagnosed with multiple, coexisting chronic conditions, and polypharmacy is common among the aging population [1, 2]. According to the World Health Organization [3] global life expectancy has increased, and as a result, the number of older people needing advanced medical care in their homes as well as in nursing homes is increasing. Health and social care services for older people have become a large and diverse sector encompassing a wide range of municipal services. In Sweden these services are divided into two separate organizations: health care managed by registered nurses and social care managed by non-licensed staff even though some have medical training. In Sweden, municipal care is largely the result of a tax-funded system in place to ensure that everyone has equal access to services, but there are challenges, including funding, quality, and efficiency. In Sweden, a first-line registered nurse is employed in each municipal council and this organization is grounded in the regulations of the National Board of Health and Welfare (HSLF-FS 2017:37). A first-line registered nurse is responsible for ensuring that all residents receive safe, good-quality care. This nurse has an overarching responsibility for ensuring the safety of procedures such as medication management and to support other registered nurses working in the municipality. Nevertheless, issues pertaining to medication management have become a risk factor in nursing homes because registered nurses employed in healthcare organizations are unable to visit all the residents who need assisted medication administration daily. One measure to help with this is that registered nurses have the prerogative to delegate medication administration to non-licensed staff. These employees may lack formal authority but will nevertheless have practical administration skills [4, 5]. This practice of delegation is not unique to Sweden and is practiced in several countries [6]. The delegation to administer drugs is given individually, and accepting the delegation is voluntary. The non-licensed staff are obligated to inform the registered nurses if they do not want to accept the delegation or are uncertain about how to perform the assignment [7]. Registered nurses are responsible for ensuring that non-licensed staff receive proper education so that the medication administration can be conducted in accordance with evidence-based practice. The delegated non-licensed staff are fully responsible for their performance and should be aware that the outcome quality influences patient safety [8]. Although Swedish regulations (SOSFS 1997:14) govern delegation, no general or mandatory education for the non-licensed staff is in place. When administering drugs, the non-licensed staff must follow a list of prescribed medication, assist the caretaker if needed, and sign that the drug was given. Any deviation in routine – for example, if no dose or the wrong dose was administered, or if there was a deviation in the schedule – must be reported to the responsible registered nurses. However, non-licensed staff do not always follow the regulations or report their mistakes [9]. Common medication management mistakes include prescription errors and errors involving the dispensation and administration of drugs, although the degree to which these affect the person under care varies [10–12]. Therefore, the caregivers’ good, safe management of medication for the elderly is important, as older persons are vulnerable due to their age and related cognitive and physical impairment. In addition, they are often unable to call for help when something goes wrong. The mistakes that can occur in medication management must be highlighted in order to be prevented; therefore, it is vital to further understand all the factors that may contribute to errors. Incidents are often attributed to human limitations or technical deficiencies, but it is important to understand that the risk of errors may be linked to factors in the workplace. The complexity of the errors can be difficult to see at first glance; and in addition, it is often easier to blame an individual than the organization [13]. As in other areas, the personal responsibility approach is the dominant tradition in medical care, but the approach has serious disadvantages [14] and may hinder the development of safe healthcare institutions [13]. The Swedish Man, Technology, and Organization concept (MTO) can be used as a method for systematically analyzing events in which the three components man, technology, and organization are given equal importance. The MTO concept as a field of knowledge comprises the knowledge needed to answer questions about why people act wrongly and how people are affected by surrounding factors [14]. The MTO concept was primarily developed to improve nuclear power plant safety, but over time, has acquired a wider application. The Swedish National Board of Health has in its beginning also used the concept when investigating reported cases of care damage [14]. Staff working in the health and social care sector have important knowledge and information of the risks they experience in their daily work. Hence, they may be an important resource for identify and to prevent risks before they do harm. Drawn from narrative reports about risk factors in drug administration, from a preventive perspective, the staff’s perceived risks are important for improving safety. Information about medication-related issues encountered on the workplace may shed light on some hidden causes to the errors.

## Methods

### The aim

The aim of this study was to explore perceptions of errors connected to medication management in nursing homes by exploring the perspective of first-line registered nurses, registered nurses and non-licensed staff involved in the care of older persons.

### Design

This study was conducted prior to the outbreak of the Covid-19 pandemic in 2020 and a qualitative deductive research approach was used based on semi-structured interviews with first-line registered nurses, registered nurses and non-licensed staff involved in medication management in nursing homes.

### Setting

Nursing homes are recommended when an older person requires staff to be available around the clock when hospital care is not needed. The staff provides medical and social care, as well as physical and occupational therapy. A general nursing home in Sweden consist of apartments that is about 30–40 square meters. It consists of combined hall, living room, bedroom, kitchenette, and sanitary space. The residents pay rent for the apartment, a nursing fee, and an additional fee for meals. This type of accommodation is a home for the elderly as well as a care environment and a workplace for non-licensed staff. Registered nurses as well as physical and occupational therapy assistants often work as consultants, which means that health and social care managers have many different aspects to consider when organizing health and social care in nursing homes.

### Participants

The author contacted the managers of health care and the social care services in seven municipalities in southern Sweden to obtain their approval for the study. The aim was to include a diverse range of regions and demographics. The managers were informed about the study at staff meetings, and the staff who were interested in participating in the study contacted the author. The data is based upon a total of 21 interviews, which included first-line registered nurses (7), registered nurses (7) and non-licensed staff (7). To be included, all informants were required to have experience of medication management and should have at least 3 years’ experience of elderly care in nursing homes. The first-line registered nurses should also have at least 1 year of employment in the position. All non-licensed staff should have the delegation to administer drugs. The informants were chosen based on the inclusion criteria, and the first person from each group who volunteered was included. Participants worked at seven nursing homes with one registered nurse and one non-licensed staff per home. The participants demographics are presented in Table 1.

**Table 1:** Characteristics of participants: First-Line Registered Nurses, Registered Nurses, and Non-licensed staff (*N* = 21)

	First-line Registered Nurses	Registered Nurses	Non-licensed staff
**Gender (number)**			
**Male**	0	1	1
**Female**	7	6	6
**Age median, (range) years**	51 (42–61)	47 (35–61)	42 (27–64)
**Working experience in elderly care at interview, years, median (range)**	5 (3–12)	9 (3–21)	12 (4^′^–25)

### Data collection

With the purpose of improving the interview technique [15], pilot interviews were conducted with one participant from each of the three occupational groups – one first-line registered nurse, one registered nurse and one non-licensed staff member. The participants were informed that their interviews were preliminary and would not be included in the study. All interviews were performed as dialogues with open-ended questions and included topics concerning views on medication management in nursing homes in general and in relation to risk situations in particular. The open dialogues gave the interviewer the freedom to make deviations in the flow of the interview and no strict guide was used. The informants chose to be interviewed at work, and at each interview, the author and the informant were seated facing each other to facilitate good social interaction. All the interviews began with the question, “What does safe drug management mean to you?” Follow-up questions were then put forward with the purpose of deepening and increasing the detail of the responses. The interviewer was familiar to the care context, which is regarded as important when posing relevant follow-up questions based on the immediate understanding of what the informants were trying to express. After each occasion, the author wrote a summary of the discussion as a which was helpful when analysing the data from tape recordings. The interviews lasted between 46 and 62 min and were transcribed verbatim into a total of 191 pages. The transcribed interviews were then coded.

### Data analysis

A deductive qualitative content analysis was used to analyze the data [16] since the purpose was to produce a systematic category system on an earlier model, in this case the MTO concept [14]. The MTO concept is well described in the literature and was considered appropriate to use in this study to analyze active failures and latent conditions due to medication management [14, 17]. The focus of the analysis was on the interaction between the three components rather than on the components themselves, with an emphasis on what the system does rather than what it is. The MTO concept can be one way to show things that go wrong, and how and why they go wrong [18]. Two sets of the data were analyzed separately by the first and second author. The analysing process started by making notes in the margin with words, theories, or short phrases that summarize the content of the text known as open coding. After the transcription, the interviews were read through in their entirety and bearing units were identified which were condensed, coded, and sorted into subcategories based on similarities. This procedure was repeated several times. The subcategories were compiled into eight categories and then sorted in accordance with MTO concept areas [14]. The authors compared and discussed the codes, subcategories, and categories until consensus was reached to make the analysis process more rigorous and to reduce the element bias [16].

### Ethics approval and consent to participate

Present study and all methods were carried out in accordance with relevant guidelines and regulations addressed in the Declaration of Helsinki, adopted by the 18th WMA General Assembly, Helsinki, Finland, June 1964. The project also followed local ethical guidelines set by Malmö University, and ethical approval of the study was obtained from the local ethical committee, Ethics Council for Care Sciences (VEN 14-12), located at Lund University. All informants and their managers were informed about the study both verbally and in writing. The informants gave their written and verbal consent to participate before the interviews. Before the inclusion of informants, an ethical consideration was made about including informants from the same workplace. The ethical considerations dealt with the possibility of creating discord between staff, and at the same time, avoided fears of answering some sensitive questions about errors or conformity in answers. The informants participated voluntarily and could end their participation at any time. All the handling of data were confidential and respected ethical principles.

## Results

**Fig. 1:**
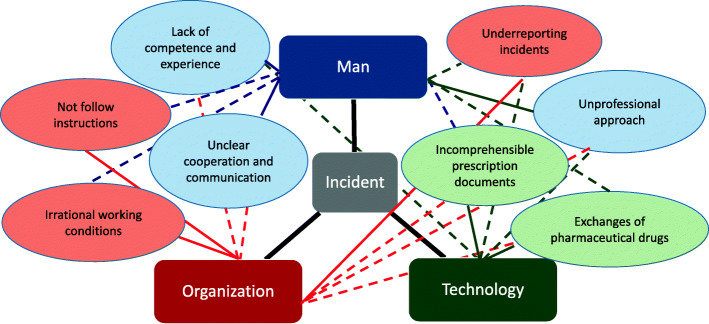
Overview over targeting errors in relation to medication management in nursing homes

The categories from the analysis were arranged according to the Man, Technology, and Organization concept (MTO) and showed that medication management – particularly the administration of drugs – was perilous in nursing homes. The informants gave many examples when safety was insufficient (Fig. 1). The informants said that mistakes in the medication administration process not only dependent on the individual but also on the working conditions. Furthermore, deficiencies were also found in the tools to facilitate and secure the medication administration process. In the interviews, it became clear that medication management errors are complex and there is generally no simple explanation for the mistakes that are made. Registered nurses and non-licensed staff work with different regulations and with different systems of documentation, which sometimes make communication and collaboration problematic. The registered nurses are not the managers of the non-licensed staff, which was emphasized as a contributing factor for mistakes. Each category in the text is reinforced with statements from the informants, for example, first-line registered nurses (FRN), registered nurses (RN) and non-licensed staff (NS).

### Man – the human factor

All the informants emphasized the human factor as a prominent reason for mistakes in medication management. They mentioned that it sometimes goes wrong because health and social care workers lack proper knowledge and skills or does not cooperate or communicate with each other. They mentioned that bad manner and unprofessional approach below or contrary to the standards courses incidents throughout the medication administration process. The categories belonging to the Man part of the MTO concept are presented below.

#### Lack of competence and experience

All the informants expressed the need for a more comprehensive delegation training program that provides sufficient knowledge to enable delegated NS to handle drug administration, not only in a correct manner but also from a safety perspective. The informants’ opinion was that discussions about drug administration issues and consequences were lacking in the training program to obtain delegation. The FRN and RN agreed that the technical part was adequate but emphasized the lack of knowledge of how anatomy and physiology relates to pharmacodynamics and furthermore of diseases caused by medication. If the NS do not understand and interpret the well-being of the patients in relation to treatment, it could jeopardize the caretaker’s health. The RNs considered this aspect important and expressed that this content should be included in a training program to certify that NS conduct safer assessments. Even though the RN is responsible, the NS must assess the risks when administering drugs. All the informants emphasized that reasoning about risks related to drug administration must be added to a training program.

“If the caregiver becomes dizzy or feels ill, gets a rash, or whatever, it is not obvious that the care staff relate these symptoms to drug treatment because their task is to look at the prescription list, hand over the drug and sign” (FRN 2)

#### Unclear cooperation and communication

Insecurity about the cooperation of the different aspects of drug management in nursing homes emerged. There was an agreement on the RNs responsibility to pass on knowledge, and the responsibility for NS to ask for help when needed. However, RNs were unsure of whether the NS communicated a need for help or if they had other questions about specific drugs that were never asked for. The NS also emphasized that, even though they belonged to the same organization, they did not always communicate or co-operate with each other. Mistakes were made when handing over drugs because there were misunderstandings about whether drugs had been administered or not, or confusion about who should do what within the team:

“It is my responsibility to provide an awareness of what it means to have a delegation and to hand over drugs from a safety perspective. But then, it is the delegate's responsibility to complete what they have learned.” (RN 4)

#### Unprofessional approach

Carelessness, difficulties in understanding responsibility issues, and not taking the task medication administration seriously were subject areas that were ventilated. Even though knowledge was available, a professional approach could be lacking. It emerged that NS sometimes did not control dosages on drug lists and chose the wrong drug from the pill organizer. It also happened that tablets disappeared and that someone later found them in the caretaker’s room. Errors were made even though NS are trained to safely hand over drugs and are informed about the importance of a written sign that shows that the drugs were administered. Informants confirmed that not controlling the number of tablets against the prescription was the most common fault. An already-administered drug could be offered to the residents when no controls were made. Changes in prescriptions were not noted, and previous prescriptions were taken for granted. The risk was even greater if NS relied on themselves to remember prescriptions or when colleagues were trusted with newly prescribed drugs without any supervision. In addition, the RN also admitted that they made mistakes. They sometimes forgot to write down new drugs on the signing list or else wrote it carelessly so that it was difficult to interpret.

“When you go to the same caretaker day in and day out, the drug handling becomes a routine – because you know how many tablets the caretaker should have, and then it almost goes by itself. You have the prescription list in mind.” (NS 1)

### Technology – tools and substitutes

A drug prescription ought to be a living document that must be communicated, updated, and reviewed regularly; however, that is not always the case. Replacement drugs are not the main issue, but the many different products may lead to difficulties in finding the correctly prescribed drug, especially if one has to read the small print on many different drug labels. The following categories outline how the administration of medicine can become hazardous and are presented below.

#### Incomprehensible prescription documents

After introducing a new type of prescription cards, reported mistakes due to misreading of the cards increased. The prescription cards may contain information about prescriptions from several physicians with several different prescription dates. There may also be more than one prescription card for each caretaker. Informants stressed that handwritten changes on cards could be unclear, and they were especially critical of when temporary drugs were prescribed or discontinued. FRNs as well as NS noted that it is the RNs obligation to make sure that the prescription cards are legible. When cards are unclear, the RN must rewrite the prescriptions, but by this action, there may be a risk for misspelling. Another hazard that was emphasized was when caretakers had two medication systems, the pill organizer and Apo dose (management of drugs by a pharmacy), and the NS only identified one of the medication systems.

“If a drug has been deleted, we have to read in many places to check that the drug really is removed. Sometimes it can be a bit tricky if you are not experienced in reading the prescription list.” (NS 7)

#### Exchanges of pharmaceutical drugs

The informants emphasized that incidents regarding replacement drugs became more and more common after a new legislation of pharmaceutical benefits in 2002 was introduced. One generic drug substance may have many different product names, which makes it especially difficult when the residents have temporary prescriptions or when they have been recently discharged from hospitals. NS did not always know the differences and similarities between different drugs, which led to several mistakes and was a common problem in their everyday work.

"... another kind of tablet that has been exchanged for another, and then they sometimes write that they didn't have the tablet at home, but they do not write the new name of the drug." (NS 3)

### Organization – within and between

Organizational deficiencies can cause errors in medication management. The categories presented below concern errors due to the lack of instructions, deprived working conditions, and underreported incidents.

#### Not following instructions

The RN informed about a demand from the social care service managers that all NS need a delegation to administer medication. The informants explained that newly employed social care staff felt a pressure to quickly accept and obtain delegation to relieve the workload of their colleagues. The FRNs stated that there was a risk that the RNs did not explain why they delegate and forgot the purpose of the training. They also noticed that, for some RNs, delegation became a purely administrative task, but somehow, they still followed the guidelines. The RNs emphasized difficulties in being updated and that it was not always easy to interpret the guidelines of the Swedish National Board of Social Security about the delegation process. It emerged in the interviews with the RNs and NS that social care workers not trained in delegation sometimes handed over drugs due to lack of staff and stressful working conditions, even though it contravened against existing regulations. Other organizational errors were that caretakers got their medication in other places than in their own room or that drugs were handed over to the wrong person. It was not always the case that NS stayed in the room to ensure that the caretaker took their medicine.

“Unfortunately, just because a non-licensed staff has been hired, and their manager asks for delegation, I give them delegation. I think that we should require an interest and a general competence and that they have a good approach and attitude.” (RN 6)

#### Irrational working conditions

Non-licensed staff with delegation were sometimes prevented from giving the drug at certain times, for example, if an incident recently had occurred or they were interrupted in the task. This led to distraction and, for example, forgetfulness if they had handed over the drug or not, counted the tablets, or signed the prescription cards. The non-licensed staff also expressed stress and concern because they explained that the time schedule for performing delegated tasks was too short. Occasionally, errors were discovered, and the caretaker got their medication, but later than prescribed. This may result in doses being given too closely in time, and as a result, the recipient’s drug concentration could be too high. When new employees were hired as substitutes during holidays or to replace sick regular employees, then the workload was higher. The substitutes often had little or no care experience. The FRNs and the RNs agreed that there were individuals among the NS and social care workers who were not interested in working in nursing homes. In addition, if the substitutes did not understand the importance of complying with the regulations, the RN believed that the risk of mistakes increased.

"I do not think that the managers always look for how many of the staff in duty have a delegation … there has been certain weekends that there was only one staff member who was delegated. And when I was on a break, there was no one … " (NS 6)

#### Underreporting incidents

The informants agreed that an under-reporting of incidents related to medication management could happen. When explaining their views, the FRNs indicated that the NS did not report events to the RNs due to lack of time, and that they did not want to write reports during their spare time. Events that occurred near the end of a work shift were most at risk of not being reported. RNs and NS informed about how incidents were not reported when staff forgot to sign the prescription cards or when prescription times were delayed. The NS and RN reported that severe mistakes and mistakes regarding medication (other than warfarin and insulin) were generally not reported based on their own judgements. The interviews further revealed that NS lack knowledge of how to write a proper incident report and that reported events did not always led to changes in routines or changes in operations to the desired extent. For actions to be made, the mistakes had to be repeated. The RNs stated that their working conditions were pressured and that they did not have time for follow-up, analyze, or report incidents to an extent they would have liked. This was well known by the FRN.

“I do not think that the registered nurses as a collective are acting consistently on medication errors. However, incident reports are not written to the extent that they should be, and the registered nurses do not always conduct a correct analysis and follow up the incidents in the way that I think they should do. If I ask them, they say there is a lack of time.” (FRN 7)

## Discussion

Incidents due to errors in medication management process in nursing homes are unfortunately a serious problem, and this study shows an ongoing complex picture of multiple risks there deficiencies in human, technological, and organizational areas overlap. Even if the intentions by first-line registered nurses, registered nurses, and non-licensed staff were good, some factors in the organization made it difficult to uphold a safe medication administration process due to lack of both staff, time and proper delegation procedures. Tools as prescription lists and devices were also pointed out as weak spots. The study shows the important to become informed about the concerns and situations in relation to underlying factors with the help of a systematic approach. Therefore, when a malicious event happens, the most important thing is to arrive at why and how the defence against this event broke down [13, 14]. Counteractive measures should therefore be based on a change in working conditions and technological tools rather than look for scapegoats. Thus, safe care can never be based on the assumption that staff do not make mistakes and if older persons in nursing homes are cared for by unskilled staff, the situation may be dangerous. Even so, if only one staff member lacks knowledge, this gap might be compensated by the sufficient competence of other personnel within the care team [18, 19]. Present study showed that non-licensed staff were sometimes careless with medication administration and their awareness of risks seemed to be generally low or forgotten. According to our opinion the most unsafe situation is when non-licensed staff are overconfidence in being able to memorize prescriptions and do not make necessary controls and thereby jeopardize the elderly persons life. This might be avoided if the registered nurses had time to support and supervise the non-licensed staff around the clock 7 days a week. In Sweden, the delegation of drug administration, was initially introduced to minimize the overall number of staff caring for the elderly, is now used to compensate for nurses who only work during the day on weekdays. When time schedules are pressed and working environments are stressful, it is difficult for non-licensed staff to find a registered nurse to consult with at nights or at weekends.

### The interplay between the organization and staff

The non-licensed staff mentioned disturbances while working with medication administration and the shortage of delegated and non-delegated staff as problematic. This finding is also confirmed in other studies [5, 20], and one of the greatest challenges of welfare is to recruit and retain staff in elderly care with the right knowledge and skills. Therefore, delegation has become more as a priority of the organization than a priority of safety. This stands in strong opposition to the regulations of The National Board of Health and Welfare (SOSFS 1997:14), which stipulates that the decision to delegate must not be based on solving staffing problems. A delegation must always be in the best interest of the caretaker and not performed simply to save time or money. We argue that a delegation process demands a thorough and accurate evaluation of the staff involved, and the first-line registered nurses have a duty of care and a legal liability to the elderly persons by setting the standards for delegation [21–23]. A first-line registered nurse must ensure that medication management has been appropriately delegated, and an appropriate level of supervision opportunity and mentorship also is available after the delegation procedure. Even so, informants in present study highlighted that the delegated staff were often unaware of the purpose of an elderly person’s medication and these eventual side effects, and thereby unable to detect serious symptoms or changes in the health due to drugs. When errors due to medication administration are likely, the ability to both deflect and correct these errors depends on the individual delegated non-licensed staffs competence, when they must decide about whether a nurse or doctor needs to be contacted. The actions taken also depends on how strong the working relationship is between the different members of staff. Delegated staff have also other duties to attend to and a great responsibility for all the resident’s health and social care. Education, training, and transforming knowledge into practice, including safety aspects, are therefore essential for non-licensed staff which is also addressed in other studies [20, 23, 24]. Therefore, we claim the need of a national consensus in a medication management training program for non-licensed staff, and the importance of time-limited delegation of drug administration with new tests to avoid blindly following routine and to have their competence updated. The registered nurses must follow up safety issues regarding medication management on a regular basis, and proactive face-to-face communication on safety issues occurring mainly during daily work is not enough. One measure could be by discussing clinical situations with the whole care team, including with first-line registered nurses and the managers of healthcare and social care services. By including managers, safety as a topic could be emphasized in the diverse organizations. A shared and reflective understanding of possible solutions of unsafe situations may increase the organizational learning.

### Technology handled by staff within and between organizations

Complex medication regimens delivered by delegated non-licensed staff combined with insufficient exchange of information and communication between staff in the two different organizations, can place elderly residents at risk for negative health consequences. In addition, systems that shall support and ensure safe medication management were in present study also looked upon as sensitive to error. To achieve satisfactory and safer tools and systems it would be better to let the staff in the organization who shall use the tools participate in the design process. Moreover, shortcomings and flaws in handling prescriptions, documentation and drugs systems, were particularly prone to errors which has been confirmed by others [25, 26]. The use of handwritten notes in prescriptions cards were additional risks, which were especially obvious when replacement drugs were used, which could lead to a confusing of drugs. Methods to secure a correctly filled-in prescription card are needed and recommended, and new technology must be developed to make it easier to act correctly. Though, systematic work on medication safety by standardized prescription cards has shown that the number of incorrect prescriptions can be reduced [27].

### Reporting deviations is fundamental regardless of framework

Important to all safety work is that all incidents are reported and analyzed so preventive actions can be taken. However, this study revealed an underreporting of incidents in terms of near misses or unsafe conditions due to medication administration which has also been shown in other studies [28, 29]. Explanations for this could be that staff are not aware of what kind of events should be reported, or the organization’s culture of safety is not conducive to reporting incidents. By ongoing discussions with staff in both organizations about safety medication management and proactively work, is therefore greatly important. An aggravating factor emphasized in the interviews was that registered and non-licensed staff were employed by different organizations with different work rules and unclear guidelines for communication and cooperation. Still we argue that a change in culture to focus less on blame and personal responsibility might increasing the number of reports. Though, managers are aware of this problem, they must take actions towards improvement. One cause of actions no taken might due to high manager and staff turnover. If management positions often change, the risk is greater that important information about reporting incidents and prevention work will be lost [30]. Furthermore, the analyzing process and response to the staff at all levels of the organizaion is important. If no feedback analyses of reported incidents are provided, information and important knowledge about the risks and incidences will be lost. Even so, staff in health and social care have important knowledge of the risks they experience in their daily work and are therefore an important resource in preventive work.

### Limitations

A strength of this study is that the data was collected through triangulation and by seeking the greatest representativeness from the three categories of personnel that gave different perspectives on the studied subject. The similarities between some of our results that are also found in other studies strengthens the transferability of our outcome. Furthermore, the analysis of the data is transparent and documented, and quotations are used to accomplish procedural rigor. Regarding interpretative rigor, two of the researchers had special knowledge and a preunderstanding of safety aspects and one did not, which deepened the researcher’s discussion and understanding of the studied subject [31]. In this study, the qualitative interview method is used to gather data, and the non-hierarchical MTO concept was applied to categorize the data. The MTO was found to be valuable because informants pointed out that human factors, organizational aspects, and technological difficulties are integrated with each other. By identifying these factors and their connections with support of the MTO concept, staff can meet and develop a common understanding of patient safety [14]. To our knowledge, no study has yet explored the integration between man, technology, and organization in relation to medication management safety in nursing homes.

## Conclusion

Medication management in nursing homes is a complex phenomenon, and caretakers are at mercy of the staff’s ability to be aware of safety and to apply good reasoning. The incidents that occur make it essential to identify deficiencies to ensure safe medication management. The purpose for the reporting system is to change critical incidents into safe behavior in safe organizations and not to identify scape goats. In this study, the MTO concept exposed that a combination of human, organization, and technology aspects are predominantly behind mistakes, and are equally important in an incident investigation. Incidents are attributed to human limitations or technical deficiencies, but organizational shortcomings also seem to be a major factor in the risk of errors. It is the attitudes and values of managers and staff that govern and shape the workplace’s safe or unsafe culture. Furthermore, safety is related to economic reality because cutbacks in the health care and social care sectors may compromise resources for work on safety. A holistic approach that integrates human, organizational, and technology aspects into safety work entails optimized solutions to improve the caretakers’ safety.

### Suggestions for improvement of safety work in medication management

- Ensure that forms for delegation follow regulations, are based on competence, and are voluntary.
- Extend education in pharmacology, physiology, pathology, and safety related to drug administration
- Develop technological devices for the prescription and administration of drugs, where staff is a part in the processes
- Increase the number of staff to keep continuity and safety competence in the workforce
- Include safety discussions about care from an ethical and professional standpoint at every workplace meeting, including experiences of risks and incidents
- Increase co-operation in care teams and include caretakers and relatives

## Data Availability

The datasets generated and analyzed during the current study are not publicly available due to an agreement with the participants on the confidentiality of the data but are available from the corresponding author upon reasonable request.

## Abbreviations

FRN: First-line registered nurses
MTO: Man, Technology, and Organization
NS: Non-licensed staff
RN: Registered nurses
